# Visible Light-Driven SnIn_4_S_8_ Photocatalyst Decorated on Polyurethane-Impregnated Microfiber Non-Woven Fabric for Pollutant Degradation

**DOI:** 10.3390/polym16030369

**Published:** 2024-01-29

**Authors:** Zhonghui Wang, Qiang Gao, Haihang Luo, Jianming Zhao, Haojun Fan, Yi Chen, Jun Xiang

**Affiliations:** 1 Key Laboratory of Leather Chemistry and Engineering of Ministry of Education, Sichuan University, Chengdu 610065, China; wangzhonghui652@163.com (Z.W.); gaoqiang_scu@163.com (Q.G.); luohaihang0823@163.com (H.L.); jmzhao@achilleskunshan.com (J.Z.); chenyi_leon@scu.edu.cn (Y.C.); junxiang@scu.edu.cn (J.X.); 2 National Engineering Research Center of Clean Technology in Leather Industry, Sichuan University, Chengdu 610065, China

**Keywords:** polyurethane, SnIn_4_S_8_, microfiber non-woven fabric, photocatalysis, degradation, recyclable

## Abstract

In recent years, polyurethane has drawn great attention because of its many advantages in physical and chemical performance. In this work, firstly, polyurethane was impregnated in a non-woven fabric (NWF). Then, polyurethane-impregnated NWF was coagulated utilizing a wet phase inversion. Finally, after alkali treatment, microfiber non-woven fabrics with a porous polyurethane matrix (PNWF) were fabricated and used as substrates. SnIn_4_S_8_ (SIS) prepared by a microwave-assisted method was used as a photocatalyst and a novel SIS/PNWF substrate with multiple uses and highly efficient catalytic degradation ability under visible light was successfully fabricated. The surface morphology, chemical and crystal structures, optical performance, and wettability of SIS/PNWF substrates were observed. Subsequently, the photocatalytic performance of SIS/PNWF substrates was investigated by the decomposition of rhodamine B (RhB) under visible light irradiation. Compared with SIS/PNWF-2% (2%, the weight ratio of SIS and PNWF, same below), SIS/PNWF-5% as well as SIS/PNWF-15%, SIS/PNWF-10% substrates exhibited superior photocatalytic efficiency of 97% in 2 h. This may be due to the superior photocatalytic performance of SIS and the inherent hierarchical porous structure of PNWF substrates. Additionally, the hydrophobicity of SIS/PNWF substrates can enable them to float on the solution and further be applied on an open-water surface. Furthermore, tensile strength and recycle experiments demonstrated that SIS/PNWF substrates possessed superior mechanical strength and excellent recycle stability. This work provides a facile and efficient pathway to prepare SIS/PNWF substrates for the degradation of organic pollutants with enhanced catalytic efficiency.

## 1. Introduction

Wastewater has attracted much more attention around the world in recent years due to its environmental and health risks [1,2]. Organic dyes, a type of significant pollutant, are widely applied in the textile, printing, paints, leather, cosmetics, paper industries, etc. [3,4]. Most dyes are very stable in the environment and difficult to be oxidated because of their complex aromatic structures [5,6]. Additionally, it has already demonstrated that dye wastewater prevents the penetration of visible light into the aqueous system, increases the biochemical oxygen demand (BOD) level, and inhibits the photosynthesis process of aquatic plants [7,8]. Organic dyes are toxic and even carcinogenic; when dyes wastewater is directly discharged into water resources without proper treatment, it can present a serious threat to aquatic life and human health. Therefore, the dyes in wastewater must be removed before they are discharged into water resources. Given this, many technologies have been used to treat dye wastewater, including adsorption, membrane separation, ion exchange, filtration, electrochemical treatment, coagulation, and biological treatment [9,10]. However, these technologies have some drawbacks, such as a high operating cost, energy consumption, being sensitive to experimental conditions, low treatment efficiency, producing secondary pollution, etc. [11]. Therefore, it is very necessary to explore novel wastewater treatment technologies. Photocatalysis has been considered as one of the most effective and eco-friendly technologies because of its unique properties of degrading organic pollution through the utilization of solar energy, no secondary pollution, strong oxidation ability, sustainability, and simplicity of operation [12,13]. As is well known, the traditional semiconductor photocatalyst TiO_2_ has been applied in the field of photocatalysis due to its low production cost, good chemical stability, excellent photocatalytic activity, and non-toxicity [14]. However, TiO_2_ still has some drawbacks, such as a large band gap (around 3.2 eV), a small specific surface area, being prone to agglomeration, a weak photo-induced carriers separation ability, a difficulty in application to continuous flow systems, and a fast photo-induced electron–hole recombination rate, all of which hinder its practical application [15,16]. Fortunately, SnIn_4_S_8_, a typical ternary metal sulfide compound with a narrow band gap (1.77–2.35 eV), strong visible-light absorption, and high stability, has occupied much concern and been applied in photocatalysis [17,18,19]. For instance, Wang and co-workers [20] fabricated hierarchical network-like SnIn_4_S_8_ microspheres via the low-temperature co-precipitation method, which exhibited the removal efficiency with a complete degradation of methyl orange within 40 min. Deng et al. [21] prepared g-C_3_N_4_/SnIn_4_S_8_ photocatalysts by the low-temperature co-precipitation method and these photocatalysts could rapidly degrade methyl orange under visible light. In Wang and co-workers’ work [22], they employed the hydrothermal method to fabricate double-shell SnIn_4_S_8_/TiO_2_ heterostructures and found that these hybrid photocatalysts displayed 90.08% degradation efficiency for methyl orange and 94.44% photoreduction efficiency for Cr (VI) within 60 min under visible light, respectively.

Up to the present, most photocatalysts have been powder-shaped materials, which are suspended in the treated wastewater. This will inevitably bring about some difficulties in recycling and reuse. On the one hand, they are not easily separated from the treated wastewater, which can result in secondary pollution to the environment [23,24,25]. Some methods have been used to separate the photocatalysts from the solution, including centrifugation, filtration, precipitation, etc. However, their separation operation procedures are complex and time-consuming. On the other hand, the aggregation and loss of powder-shaped materials in the recycling process could cause a decline in catalytic efficiency [26,27]. Furthermore, it is unsuitable for application in continuous flow systems [28]. Several approaches have been suggested to solve the disadvantages of powder-shaped photocatalysts in practical applications, including immobilization of powder-shaped photocatalyst in polymers, glass, ceramics, magnetic materials, etc. [29,30,31]. In Stoilova and co-workers’ work [32], they incorporated TiO_2_ into poly(methyl methacrylate) fibers by the electrospinning method and found that this type of composite could degrade methylene blue under ultraviolet light irradiation. Fu and co-workers [33] immobilized BiOI/rGO photocatalysts on Fe_3_O_4_ magnetic materials to fabricate BiOI/rGO/Fe_3_O_4_ hybrid photocatalysts. These photocatalysts could be rapidly separated from the reaction solution by an external magnet and showed 82.3% degradation efficiency for RhB after 10 cycles. In Zainal and co-workers’ work [34], they immobilized TiO_2_-Chitosan photocatalysts on the glass and found that this type of hybrid photocatalysts could decompose methyl orange under a 15 W Philips white fluorescent light source. Recently, non-woven fabric (NWF) substrate has received widespread attention owing to its non-toxicity, good floatability, unique chemical inertness as well as its facile recovery [35]. These advantages make it a promising candidate for immobilizing powder-shaped materials, easier recovery, recycling, and reuse. Dao et al. [36] fabricated a type of non-woven polyester fabric-supported Cu_2_O/reduced graphene oxide nanocomposite; this photocatalyst exhibited 96% removal efficiency for methylene blue in 120 min. Huo et al. [37] assembled macroscopic D-g-C_3_N_4_/sodium alginates/non-woven fabric by means of a two-step method of coating and cross-linking with CaCl_2_, which showed 94.3% degradation efficiency of RhB in 80 min and 97.8% degradation efficiency of tetracycline hydrochloride in 60 min under visible light irradiation, respectively. Meanwhile, this type of non-woven fabric photocatalysts could be easily recycled and retains excellent stability in the photocatalytic reaction.

Up to now, polyurethane has received a lot of attention due to its ascendant mechanical performance, favorable abrasion resistance, and superior flexibility [38,39]. In view of this, to improve the elasticity and tensile strength of non-woven fabrics, polyurethane with repeating carbamate groups (–NHCOO–) was impregnated in the fibers. Subsequently, polyurethane-impregnated non-woven fabric was coagulated by means of a wet phase inversion. Finally, after alkali treatment, microfiber non-woven fabric with a porous polyurethane matrix (PNWF) was fabricated. On account of its three-dimensional structure, high porosity, good mechanical intensity as well as its laudable chemical resistance, microfiber non-woven fabric has been widely applied in synthetic leather, wear, electromagnetic shielding, etc. [40,41,42,43]. However, there have been few reports on its applications in photocatalysis.

Recently, a microwave-assisted method has been widely used due to its unique characteristics of fast heating speed, high yields, high purity of the products, and homogenous dielectric heating [44,45]. Compared with the conventional hydrothermal method, the microwave-assisted method can shorten the reaction time from several hours to a few minutes or even seconds. Chumha et al. [46] prepared CuInS_2_ nanoparticles by the microwave heating process with a 7.5 min reaction time and discovered that this photocatalysts could degrade methylene blue and rhodamine B with 91.21% and 97.8% degradation efficiency within 360 min under visible light irradiation, respectively. Li and co-workers [47] synthesized Fe_3_O_4_/rGO composites by the microwave synthesis method. They found the size and morphology of nanocomposites could be tuned by changing the microwave reaction time. Therefore, the microwave-assisted method is expected to rapidly prepare photocatalysts and further contribute to improving the efficiency of the dye wastewater treatment.

In this work, in order to solve the disadvantages of powder-shaped photocatalysts in recycling and reuse, SIS/PNWF substrates were firstly fabricated and applied in the field of photocatalysis. First of all, SIS was synthesized by the means of a microwave-assisted method according to our previous report [48], and deposited on the PNWF substrates to assemble the SIS/PNWF substrates. Subsequently, the surface morphology, structure and optical performance of as-prepared SIS and a series of SIS/PNWF substrates were observed by scanning electron microscope, X-ray photoelectron spectroscopy, X-ray diffraction instruments, attenuated total reflection-Fourier transform infrared spectrometer, and UV-Vis-NIR diffuse reflectance spectrophotometer, respectively. The hydrophobicity of SIS/PNWF substrates can allow them to float on the solution and be applied in an open water surface. In addition, the photocatalytic performance of SIS/PNWF substrates was investigated by the decomposition of RhB under visible light irradiation. The tensile strength and recycle experiments demonstrated that SIS/PNWF substrates possessed superior mechanical strength and excellent recycle stability. Especially, after each photocatalytic reaction, the spent SIS/PNWF substrates were easily recovered from the reaction system by a nipper, which solved the disadvantages of powder-shaped photocatalysts in recycling and reuse. Thus, this work provides a facile and efficient pathway to prepare SIS/PNWF substrates for the degradation of organic pollutants.

## 2. Experiment

### 2.1. Materials

Thioacetamide (TAA, 99.0%), sodium dodecyl benzene sulfonate (SDBS), tin chloride pentahydrate (SnCl_4_·5H_2_O, 99.9%), ammonium sulfate ((NH_4_)_2_SO_4_), and indium chloride tetrahydrate (InCl_3_·4H_2_O, 99.9%) were obtained from Aladdin Chemical Reagent Co., Ltd. (Shanghai, China). Ethanol and sodium hydroxide (NaOH) were acquired from the Kelong Chemical Incorporated Co. Ltd., Chengdu, China. Waterborne polyurethane was prepared according to our previous work [49]. All reagents were used without any further purification. The sea–island bicomponent non-woven fabric compositing with polyamide-6 (PA6) and polyethylene terephthalate (PET) was obtained from the Zhejiang Meisheng Industrial Co., Ltd. (Shaoxing, China).

### 2.2. Fabrication of Three-Dimensional Microfiber Non-Woven Fabric

First, the sea–island bicomponent NWF was dipped in waterborne polyurethane with 25 wt% solid content. Waterborne polyurethane was utterly and uniformly penetrated into the NWF by pressing and squeezing with a round iron bar. Then, polyurethane-impregnated NWF was coagulated by being submerged into the ammonium sulfate solution (3 wt%) at pH 2–3 for 25 min. Subsequently, the aforementioned NWF was immersed in the 10 wt% NaOH solution at 90 °C for 90 min to remove PET. After washing and drying, the three-dimensional PNWF substrates with high porosity were fabricated.

### 2.3. Preparation of SnIn_4_S_8_ Photocatalysts

According to our previous report [48], SIS was synthesized via the microwave-assisted method. First of all, 0.6 mmol InCl_3_·4H_2_O, 0.15 mmol SnCl_4_·5H_2_O, 1.89 mmol SDBS, and 1.2 mmol TAA were dispersed in 50 mL anhydrous ethanol and continuous stirring was applied for 40 min at room temperature. Subsequently, the above-mentioned mixture was transferred to the microwave teflon vessel with an automatic application of microwave power output (0 to 950 W) and temperature sensor. Then, the microwave unit was heated at 180 °C for 20 min. After the reaction, the microwave reaction vessel was cooled down using compressed air. Finally, the yellow product was obtained and washed with deionized water and ethanol three times and freeze-dried for further use.

### 2.4. Preparation of SIS/PNWF Substrates

Firstly, a certain quantity of SnIn_4_S_8_ photocatalysts was dispersed in 50 mL deionized water. The PNWF substrate was cut into a size of 4 cm × 4 cm, was then immersed into the as-prepared SIS suspension, and shaken for 2 h at room temperature. The obtained SIS/PNWF substrates were washed in deionized water three times and then dried in an oven at 70 °C for 8 h. SIS/PNWF substrates were defined as SIS/PNWF-2% (2%, the weight ratio of SIS and PNWF, same below), SIS/PNWF-5%, SIS/PNWF-10%, and SIS/PNWF-15%. 

### 2.5. Evaluation of Photocatalytic Activity

The photocatalytic activities of a series of SIS/PNWF substrates were evaluated by degradation of RhB. A 300 W xenon lamp (CEL-HXF300, China Education Au-light company, Beijing, China) coupled with an optical cutoff filter of 400 nm was utilized as a visible light source. The distance between the light source and the sample was 10 cm. A series of SIS/PNWF substrates were immersed in 50 mL solution with 30 mg/L RhB, respectively. The aforementioned suspension was constantly stirred in the dark for 2 h to achieve an adsorption–desorption equilibrium. Then the suspension with SIS/PNWF substrates was exposed to a 300 W xenon lamp under constant stirring conditions. Subsequently, approximately 3 mL of aliquot was collected at different times and immediately filtered through a 0.45 µm filter membrane. The absorbance of the RhB was analyzed by a UV-Vis spectrophotometer with a scan speed of 600 nm·min^−1^ at 556 nm (3900H, Hitachi, Tokyo, Japan). The following equation was used to evaluate the RhB removal efficiency:

RhB removal efficiency (RE) = (A_0_ − A_t_)/A_0_ × 100%, where A_0_ represents the initial absorbance of RhB, and A_t_ denotes the absorbance of RhB at a certain reaction time t min.

### 2.6. Characterization

Surface morphologies of as-prepared SIS, NWF, PNWF, and SIS/PNWF-10% substrates were surveyed via a Cambridge CamScanCS3400 field emission scanning electron microscope (SEM). X-ray photoelectron spectroscopy (XPS) analysis of the SIS/PNWF-10% substrate was conducted on a Kratos XSAM800 system with an Al Ka. The crystal phases of SIS and SIS/PNWF substrates were observed by an X-ray diffraction (XRD) instrument (Kratos, AXIS Ultra DLD) using CuKa radiation in the two theta ranging from 10° to 80°. UV-Vis-NIR diffuse reflectance spectra of SIS and a series of SIS/PNWF substrates were performed on a PE1050+ spectrophotometer with an integrating sphere (PerkinElmer, Waltham, MA, USA) ranging from 200 to 700 nm at room temperature. Barium sulfate was used as a reference in the testing experiment. The absorbance of the RhB solution was measured by a UV-Vis spectrophotometer (3900H, Hitachi, Japan) with the scan rate of 600 nm·min^−1^ at 556 nm. Attenuated total reflection-Fourier transform infrared (ATR-FTIR) spectra of the as-prepared samples were measured by a spectrum 3 FTIR spectrometer (PerkinElmer, USA) in a wavenumber range from 650 to 4000 cm^−1^ at 2 cm^−1^ resolution. The water contact angles of NWF, PNWF, SIS/PNWF-10% substrates were measured by an optical contact angle goniometer (DSA30s, KRUSS, Hamburg, Germany) with an automatic controller and a high-speed camera using 3 μL water drops at room temperature. The tensile strength of as-prepared SIS/PNWF substrates was determined on an electronic universal testing machine (AI-7000 SN, Gotech Testing Machines Co., Ltd., Dongguan, China) equipped with a 100 N load cell with a tensile speed of 100 mm·min^−1^ at room temperature. The average thickness of each sample was measured by a GT-313-A pachymeter (Gotech Testing Machines, China) at room temperature. Every as-prepared sample was measured three times to calculate their average value and standard deviation. 

### 2.7. Regeneration and Reuse of SIS/PNWF Substrates

After each photocatalytic reaction, the spent SIS/PNWF substrates were easily recovered from the reaction system by a nipper, then washed with 100 mL DI water three times and dried at 60 °C for 8 h. The regenerated SIS/PNWF substrates were reused for the decomposition of RhB under the same reaction conditions as with the fresh SIS/PNWF substrates.

## 3. Results and Discussion

### 3.1. Morphology

**Figure 1:**
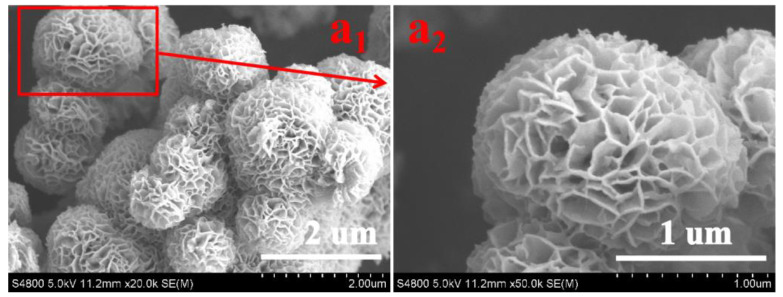
The low-magnification (**a_1_**) and high-magnification (**a_2_**) SEM images of SIS.

The surface morphologies of as-prepared SIS, NWF, PNWF and SIS/PNWF-10% substrates were observed by SEM. The pure SIS displays noticeable 3D hierarchical urchin-like microspheres with an average diameter of approximately 1–2 µm (Figure 1(a_1_)). The high magnification SEM image (Figure 1(a_2_)) further demonstrates that the urchin-like microspheres are fabricated by in situ self-assembly of the 2D disordered nanosheets, which is consistent with our previous work [48].

**Figure 2:**
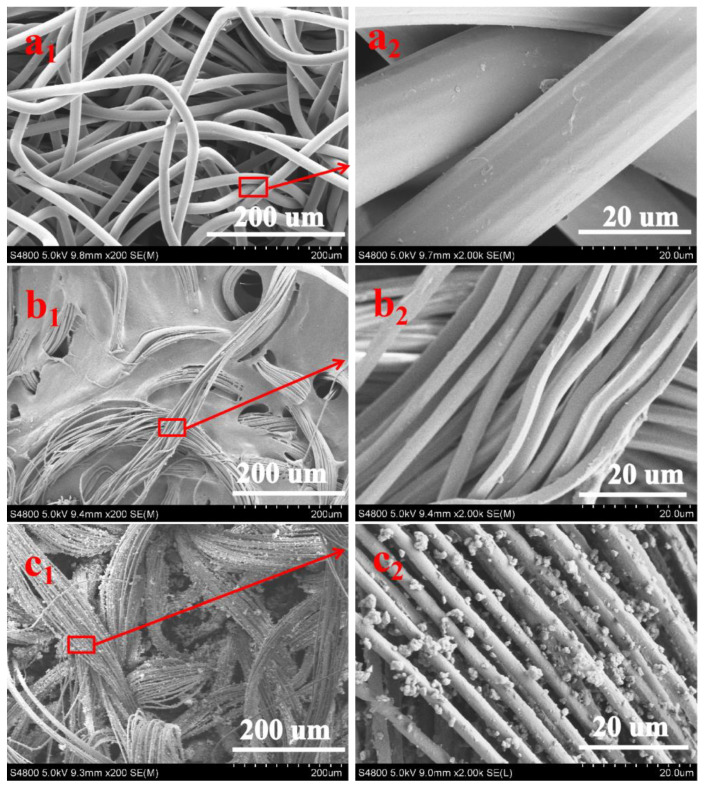
The SEM images of NWF (**a_1_**,**a_2_**), PNWF (**b_1_**,**b_2_**) and SIS/PNWF-10% substrates (**c_1_**,**c_2_**).

The SEM images of NWF, PNWF and SIS/PNWF-10% substrates are illustrated in Figure 2, respectively. For the NWF, it reveals that the average diameter of the single sea–island fiber is about 20 μm (Figure 2(a_1_,a_2_)). Figure 2(b_1_,b_2_) indicates that the individual island fiber is separated into many microfibers with a diameter of approximately 2–3 µm, which is clearly lower than that of the single sea–island fiber for the NWF. Further observation reveals that there are plentiful voids in the PNWF substrate, which can favor increasing the specific surface area of the substrates. As depicted in Figure 2(c_1_,c_2_), SIS microspheres are densely distributed on the surface of PNWF substrates. These results show that SIS/PNWF substrates were successfully fabricated.

### 3.2. Crystal Structure

**Figure 3:**
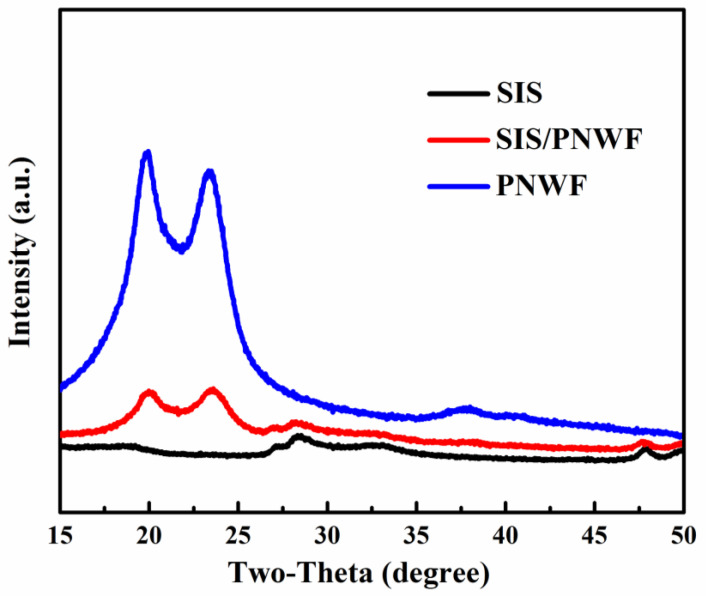
The XRD patterns of SIS, SIS/PNWF-10%, and PNWF substrates.

The XRD patterns of as-prepared SIS, PNWF, and SIS/PNWF substrates are presented in Figure 3. The five main characteristic diffraction peaks at 18.5°, 27.4°, 28.9°, 48.0°, and 49.8° can be readily ascribed to the (202), (311), (222), (440), (531) crystalline planes of the cubic phase of SnIn_4_S_8_, respectively. The peaks at two theta values of 20.8° and 23.8° correspond to the (200) and (002) crystalline planes of polyamide-6. Interestingly, the diffraction peaks of SIS at two thetas of 27.4°, 28.9°, 48.0° and 49.8° can be observed on the SIS/PNWF substrates. These results further demonstrate that SIS was successfully deposited on the surface of PNWF substrates, and the structures of the SIS and PNWF had no significant influence on the hybridization process.

### 3.3. XPS Analysis

**Figure 4:**
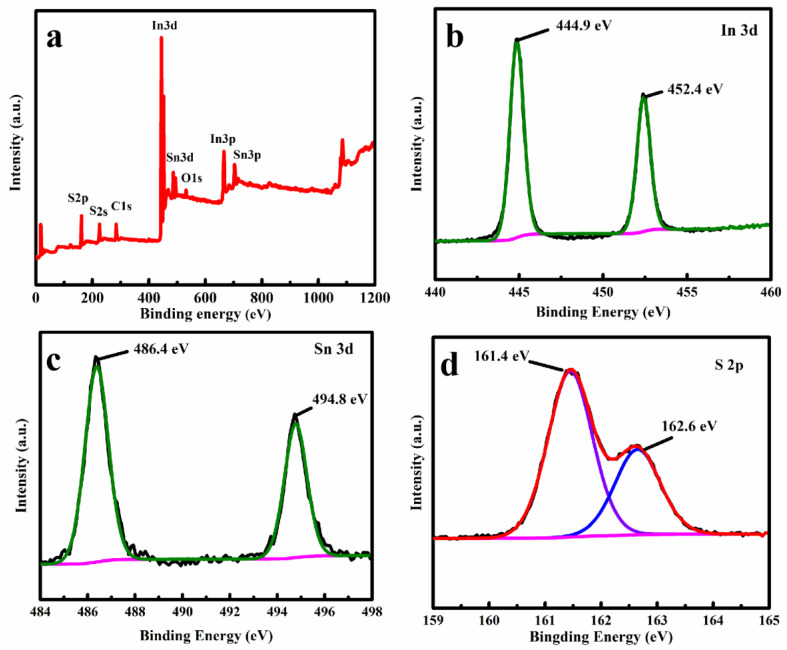
The XPS survey spectrum (**a**) and In 3d (**b**), Sn 3d (**c**), S 2P (**d**) spectra of SIS/PNWF-10% substrate.

XPS was employed to investigate the element’s composition and the chemical state of the SIS/PNWF-10% substrate. A survey spectrum clearly shows that the Sn, In, and S elements existed in the SIS/PNWF substrate (Figure 4a). The intense peaks located at 444.9 eV and 452.4 eV (Figure 4b) are attributed to In 3d_5/2_ and In 3d_3/2_ of In^3+^, respectively. As exhibited in Figure 4c, the obvious peaks of 486.4 eV and 494.8 eV can be assigned to the Sn 3d_5/2_ and Sn 3d_3/2_, respectively. This result demonstrates that Sn^4+^ exists in the SIS/PNWF substrate. As for the S 2p spectrum (Figure 4d), two apparent peaks at 161.4 eV and 162.6 eV can be assigned to the S 2p_1/2_ and S 2p_3/2_ of S^2−^, respectively. In other words, the above results further confirm the formation of SIS/PNWF substrates.

### 3.4. UV-Vis Diffuse Reflectance Spectra

**Figure 5:**
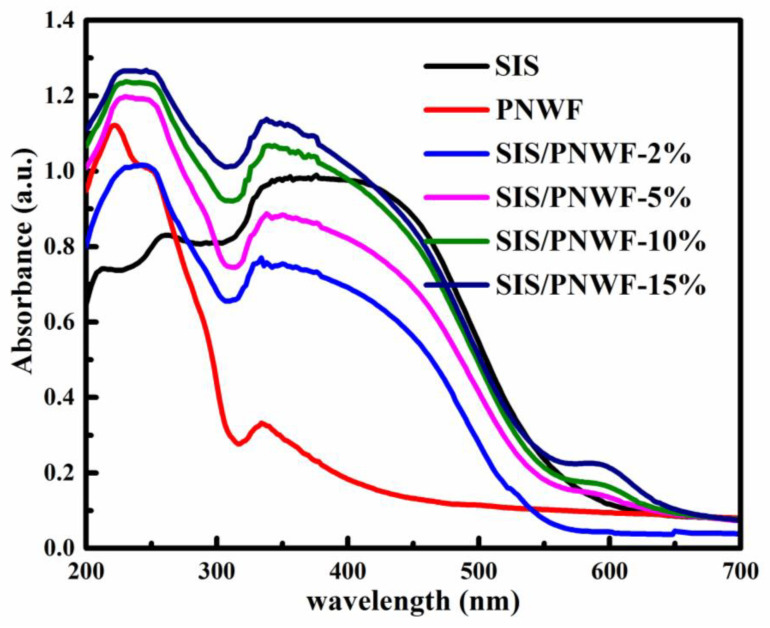
The UV-vis diffuse reflectance spectra of SIS, PNWF, SIS/PNWF-2%, SIS/PNWF-5%, SIS/PNWF-10%, and SIS/PNWF-15% substrates.

The optical absorption properties of the PNWF and a series of SIS/PNWF were determined by UV-Vis diffuse reflectance spectrometer with a wavelength ranging from 200 to 780 nm at room temperature. As denoted in Figure 5, PNWF substrates display no obvious absorption at a wavelength greater than 320 nm. After SIS was deposited on the surface of the PNWF, the absorption intensities of the SIS/PNWF in the visible light region distinctly increased, which can be ascribed to the strong absorption of SIS in the visible region. Furthermore, the absorption edges of the SIS/PNWF substrates are apparently red-shifted with the increase in SIS photocatalyst contents. These features suggested that the SIS photocatalysts endowed the PNWF with a conspicuous visible-light responsive performance. 

### 3.5. FT-IR Spectra

**Figure 6:**
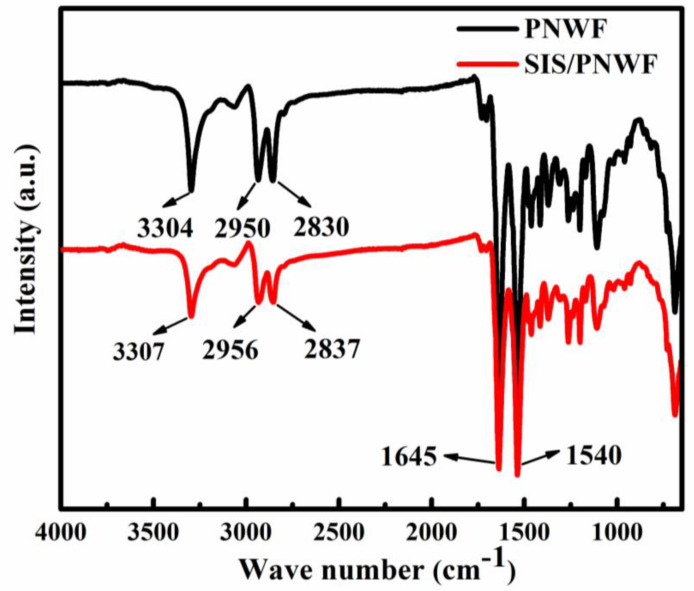
FTIR spectra of PNWF and SIS/PNWF−10% substrates.

ATR-FTIR spectroscopy was applied to survey the surface chemical structure change in PNWF and SIS/PNWF-10% substrates. As illustrated in Figure 6, the characteristic peaks are at 3304, 1645, and 1540 cm^−1^, which is consistent with υ_N–H_, υ_C=O_ and the combining absorption of both δ_N–H_ and υ_C–N_, respectively. The prominent peaks at 2950 and 2830 cm^−1^ correspond to the C–H stretching vibration for -CH_3_. Further observation reveals that the spectrum of the PNWF is akin to that of the SIS/PNWF substrate. Therefore, the FT-IR spectrum indicates that the structure of the PNWF is not affected by the incorporation procedure of SIS.

### 3.6. Wettability

**Figure 7:**
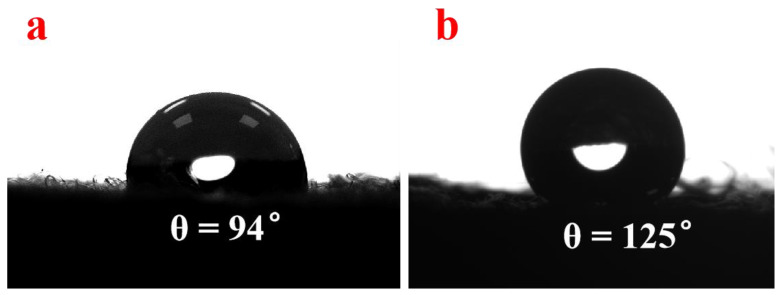
The water contact angles of PNWF (**a**), SIS/PNWF-10% (**b**) substrates.

Wettability is the crucial factor affecting the practical application. In order to compare the difference in the wettability of as-prepared samples, the water contact angles of NWF, PNWF and SIS/PNWF-10% substrates were measured with a contact angle goniometer with an automatic controller and a high-speed camera. In this experiment, the water drops can rapidly spread and wet the NWF substrate, while water drops on the PNWF and SIS/PNWF-10% substrates can remain on the substrates surface for a while. As presented in Figure 7, the water contact angles of the PNWF and SIS/PNWF-10% substrates are 94° and 125°, respectively. This result demonstrates that SIS loading can change the wettability of the PNWF substrate surface. It can be explained that hierarchical urchin-like microspheres composed of the disordered nanosheets can trap a lot of air in their pores, resulting in a certain hydrophobicity of the SIS/PNWF-10% substrate [50]. On account of this hydrophobic performance, the SIS/PNWF-10% substrate can also be applied in wastewater treatment on an open surface such as a lake. 

### 3.7. Photocatalytic Properties of SIS/PNWF Substrates

**Figure 8:**
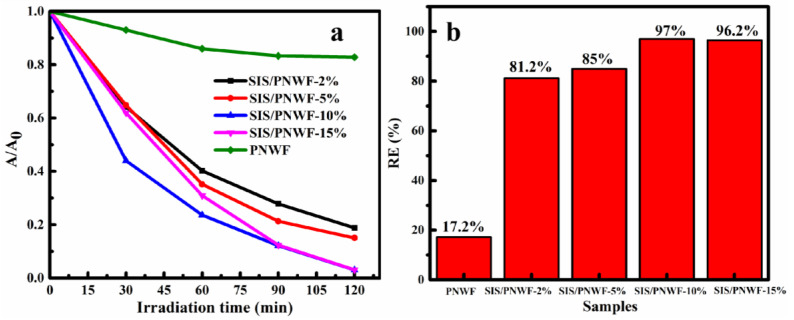
Photocatalytic activity (**a**) and photocatalytic degradation efficiency (**b**) of PNWF, SIS/PNWF-2%, SIS/PNWF-5%, SIS/PNWF-10%, and SIS/PNWF-15% substrates for RhB under visible light irradiation.

The photocatalytic activity for RhB over a series of SIS/PNWF substrates was estimated under visible light. Compared with the PNWF, the SIS/PNWF substrates show enhanced photocatalytic performance (Figure 8). Just as was predicted, the photocatalytic activity of the SIS/PNWF substrates is clearly affected by the SIS content. It is clear that the RhB degradation rate gradually rises from 81.2% to 97% when the SIS photocatalysts content increases from 2% to 10%, whereas the degradation efficiency of RhB descends when the dosage ratio of SIS photocatalysts further increases to 15%. It can be inferred that an excess of photocatalysts could increase light scattering and bring down the absorbance, resulting in a decline in the degradation rate of the contaminants [51]. Therefore, the photocatalytic activity of a series of as-prepared SIS/PNWF substrates first increased and then reduced with an increase in the SIS content, indicating that the optimized weight ratio of SIS to PNWF was 10%. The enhanced photocatalytic activity of the SIS/PNWF-10% substrate should be attributed to the successful loading of SIS, the strong light absorption, and the inherent hierarchical porous structure of the PNWF substrate.

### 3.8. Reusability and Stability of the SIS/PNWF Substrates

**Figure 9:**
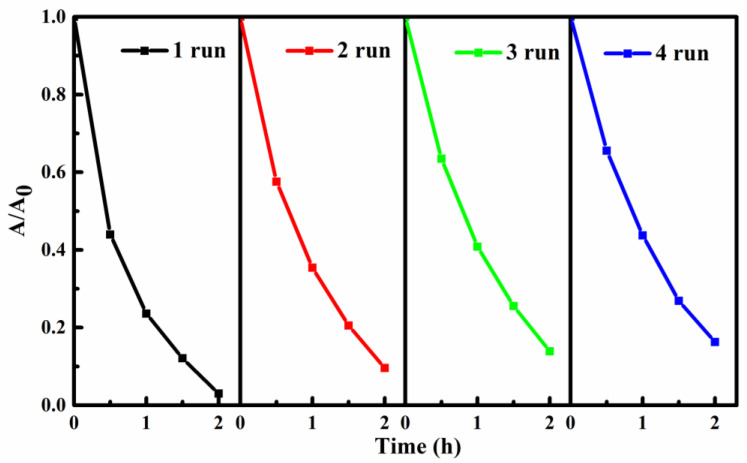
Photodegradation reusability of RhB over SIS/PNWF-10% substrate.

Generally, the reusability and stability of photocatalysts are key factors affecting their practical application. In this work, the reusability and stability of SIS/PNWF substrates were studied. After each photocatalytic reaction, the SIS/PNWF substrate was pulled out from the reaction system by a nipper, then washed with DI water several times and dried at 60 °C for 8 h. As shown in Figure 9, the removal rate of RhB can reach up to 97% within 2 h under a visible light irradiation. After running four cycles, the degradation rate of RhB can still maintain 84%. The results indicate that SIS/PNWF substrates have good recyclability and stability. A slight decrease in the photocatalytic activity of the recovered SIS/PNWF substrates may be explained by the fact that a small amount of SIS photocatalyst could be lost in the process of consecutive recycling experiments. 

### 3.9. Tensile Strength of the SIS/PNWF Substrates

**Figure 10:**
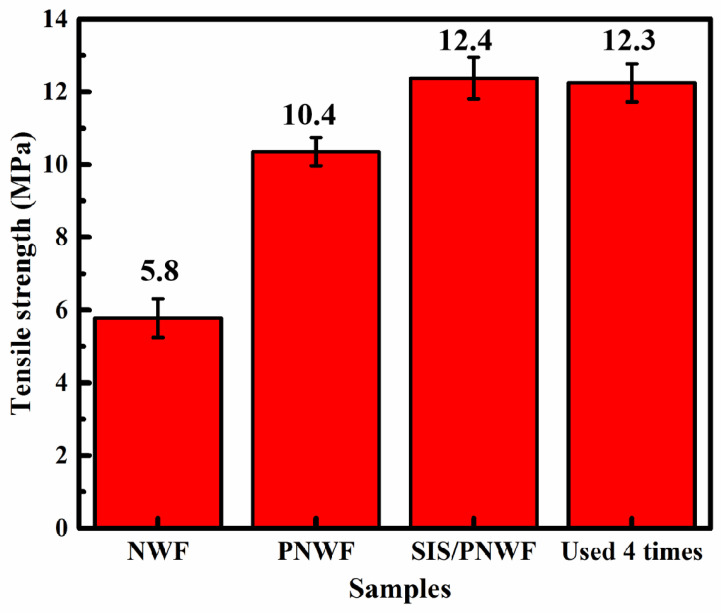
Tensile strength of NWF, PNWF, SIS/PNWF-10%, and SIS/PNWF-10% substrate used 4 times.

Mechanical performance is the key factor affecting the stability of the materials [52]. The tensile strength of the NWF, PNWF, SIS/PNWF-10%, and SIS/PNWF-10% (used four times) substrates was evaluated by an electronic universal testing machine with the tensile speed of 100 mm·min^−1^ at room temperature. As depicted in Figure 10, the tensile strength of the PNWF substrate with the value of 10 MPa is greater than that of the NWF substrate, which indicates that polyurethane is beneficial for improving the tensile strength of the PNWF substrate. Interestingly, after depositing SIS photocatalysts on the PNWF substrate, the SIS/PNWF-10% substrate showed enhanced tensile strength with a value of 12.4 MPa. This result can be interpreted as indicating that SIS photocatalysts are conducive to enhancing the tensile strength of the PNWF substrate. Moreover, after running four cycles, the SIS/PNWF-10% substrate still maintains excellent tensile strength with the value of 12.3 MPa. Thus, these results indicate that the SIS/PNWF-10% substrate possesses superior tensile strength.

## 4. Conclusions

In summary, microfiber non-woven fabrics with a porous polyurethane matrix were fabricated by impregnated polyurethane, coagulation, and alkali treatment procedures. SIS was prepared by a facile microwave-assisted method and deposited on polyurethane-impregnated NWF substrates. The optimal SIS/PNWF-10% substrate displayed enhanced photocatalytic activity with a 97% degradation rate to RhB within 2 h under visible light irradiation. The enhanced photocatalytic activity of the SIS/PNWF-10% substrate should be attributed to the successful loading of SIS, the strong light absorption, and an inherent hierarchical porous structure of the PNWF substrates. Additionally, the SIS/PNWF-10% substrate showed excellent reusability and stability, and superior tensile strength. The hydrophobicity of the SIS/PNWF substrates could enable them to float on the solution and be applied on an open-water surface. This work provides a facile and efficient pathway to prepare SIS/PNWF substrates for the degradation of organic pollutants.

## Data Availability

The data presented in this study are available on request from the corresponding author.
